# Prevalence and predictors of HIV-related disability among people living with HIV in Nigeria

**DOI:** 10.4102/sajp.v80i1.2001

**Published:** 2024-07-30

**Authors:** Temitope S. Oladejo, Hellen Myezwa, Adedayo T. Ajidahun, Sam Ibeneme

**Affiliations:** 1 Department of Physiotherapy, Faculty of Health Sciences, University of the Witwatersrand, Johannesburg, South Africa; 2 School of Therapeutic Sciences, Faculty of Health Sciences, University of the Witwatersrand, Johannesburg, South Africa; 3 Department of Medical Rehabilitation, Faculty of Health Sciences, University of Nigeria, Enugu, Nigeria

**Keywords:** HIV, disability, activities of daily living, health-related quality of life, prevalence, predictors

## Abstract

**Background:**

People living with human immunodeficiency virus (PLWH) live longer, but experience human immunodeficiency virus (HIV)-related comorbidities and disabilities that lower their quality of life. Understanding the prevalence, risk factors, and disability patterns is crucial for tailored interventions.

**Objectives:**

To explore the prevalence and predictors of HIV-related disability among PLWH in Nigeria.

**Method:**

This cross-sectional survey involved 385 PLWH, exploring demographic data, HIV history, recent symptoms, disability (measured by the WHO Disability Assessment Schedule–WHODAS 2.0). Descriptive statistics summarised the data, all variables were entered into univariate and multivariate regression models. IBM SPSS 25^®^ was used for all analyses at a 95% confidence level.

**Results:**

The prevalence of disability among PLWH was 39.5%, the mean age was 42.2 ± 10.43 years, and 73% of the participants were females. Factors significantly associated with disability were marital status (*p* = 0.009) and level of education (*p* = 0.001).

**Conclusion:**

The study reveals a prevalence of disability (39.5%) among PLWH on antiretroviral therapy (ART), emphasising the need for tailored interventions considering socio-demographic factors. Continuous screening, risk identification, and effective management strategies are imperative, recognising disability as an indicator of health and quality of life.

**Clinical implications:**

With PLWH experiencing increased life expectancy, the study underscores the need for an informed patient-centred approach to care, recognising the specific challenges faced by PLWH in Nigeria and guiding the development of targeted interventions to enhance both functional outcomes and overall well-being.

## Introduction

The development of disability among people living with human immunodeficiency virus (PLWH) has become an increasingly significant global health problem. Of the 38.4 million PLWH globally, 67% live in sub-Saharan Africa (Joint United Nations Programme on HIV/AIDS [UNAIDS] 2021). Nigeria has an estimated human immunodeficiency virus (HIV) prevalence of 2.1% (Onovo et al. 2023), accounting for two million of the nation’s population. Of Nigeria’s estimated population of 200 million, approximately 25 million live with one or other form of disability (Haruna 2017). Human immunodeficiency virus and/or acquired immunodeficiency syndrome (AIDS) ranks among the top causes of disability-adjusted life years (DALYs), second only to malaria (The Lancet 2022). In line with the 95-95-95 vision of UNAIDS (Frescura et al. 2022), Nigeria’s proportion of people with access to antiretroviral therapy (ART) has increased over the years; this implies that PLWH have an extended life expectancy, thereby living longer lives (Federal Ministry of Health, Nigeria & Nigeria HIV/AIDS Indicator and Impact Survey [NAIIS] 2019). However, the combination of HIV infection and the side effects of ART contributes to the increased emergence of disabilities among PLWH owing to ageing in the long-term survivors of HIV.

As people age with HIV, there is an increased likelihood of developing comorbidities, such as cardiovascular diseases, osteoporosis, chronic obstructive pulmonary disease, chronic kidney disease, and certain cancers (Crane & Drumright 2022; Pelchen-Matthews et al. 2018; Roomaney et al. 2022). The sequelae of the above comorbidities involve disabilities related to functional limitations and social participation restrictions, the types, and patterns of which may vary across socio-cultural boundaries, but these need to be properly elucidated to inform relevant rehabilitation interventions. The International Classification of Functioning, Disability, and Health offers a normative metric and conceptualises that disability may be related to impairments (fatigue, pain), functional capacities, activity limitations, and participation restrictions (WHO 2013). With HIV being varied in its impact, and disability being a multi-dimensional concept, the International Classification of Functioning, Disability and Health (ICF) (World Health Organization, 2001) model examines the dynamic interaction between HIV-related disability and the influence of contextual (environmental and personal) factors (Kietrys et al. 2019).

At the individual level, determining the specific disability caused by HIV versus the disability caused by social determinants influencing HIV exposure can be difficult and intertwined (UNAIDS 2017). Thus, to detangle this intersectionality of HIV and disability, a comprehensive approach that considers both the physiological consequences of HIV infection and the broader social context in which PLWH live should be considered. Brown et al. (2022) highlighted that the risk factors for HIV-related disability can be classified into three categories: socio-demographic factors, social determinants of health, and HIV features such as late diagnosis. These factors may cause PLWH to experience greater limitations in performing basic activities.

Numerous studies (Banda et al. 2019; Erlandson 2020; Payne et al. 2022) have explored the diverse nature of impairments and their associated functional limitations that prevent PLWH from fully engaging in activities of daily living. The severity of HIV-related disability ranges from mild to moderate to severe. The prevalence of moderate and severe disability among people living in the United Kingdom (UK) was recently reported as 70.5% and 39.5%, respectively (Brown et al. 2022). A systematic review of data among PLWH from sub-Saharan Africa reported that the prevalence of disability was high across a range of body functions and/or structures (Banks et al. 2015). According to Olaleye, Adetoye, and Hamzat (2017), 71.1% of PLWH in Ibadan, Nigeria, were found to have a mild to moderate level of disability, as measured by the WHO Disability Assessment Schedule (WHODAS 2.0). Similarly, 51.9% of the participants in a South-African study scored 2 or more on the WHODAS 2.0 scale indicating the presence of moderate level disability.

Although sub-Saharan Africa bears the greatest disease burden of people acquiring and living with HIV (Jahagirdar et al. 2021), the impact of HIV on disability in sub-Saharan Africa has not been expansively researched, and there is a dearth of literature from Nigeria. Given the diverse socio-demographic, economic, and socio-cultural factors that could impact the psychosocial wellbeing of PLWH in Nigeria and the development of disability (Ogunmola, Oladosu & Olamoyegun 2014), understanding the prevalence and patterns of HIV-related disabilities and their associated factors would help develop a contextualised management approach. Hence, the aim of our study is to determine the prevalence and associated factors of HIV-related disability among PLWH in Nigeria.

## Research methods and design

A cross-sectional descriptive study design was used in our study. The study involved PLWH recruited from seven HIV and AIDS Testing and Treatment Centres in Lagos. Eligibility for the study included being 18 years old and above, living with HIV, and attending the outpatient HIV and AIDS clinic.

### Study setting

The study was multicentred and conducted in Lagos State. This state was selected in the light of the availability of resources, the size of the country’s total population, and because it is one of the major population hotspots with evidence of a high HIV burden and unmet needs for HIV and AIDS treatment services (Lo et al. 2021). Lagos, with over 24 million residents (Obilaonu, Mohd & Norhuzailin 2023) has 31 HIV/AIDS (Lo et al. 2021) Testing and Treatment Centres spread across 20 Local Government Areas.

### Sample size

Of the estimated 2 million PLWH in Nigeria, 24 000 live in Lagos (Onovo et al. 2023). The sample size was determined by using the single population proportion formula (Bujang 2021), where the following assumptions were considered: 95% confidence interval, 51.9% prevalence of disability among PLWH (Myezwa et al. 2018) and 5% margin of error. Thus, a final sample size of 385 was calculated.

### Sampling method

For this study, seven facilities were randomly selected. Study participants were recruited using systematic random sampling at the HIV clinics.

### Data collection

Data were obtained through self-administered formats. The standardised questionnaires listed below were employed for collecting data according to the specific study objectives.

#### Socio-demographic questionnaire

The socio-demographic questionnaire used in our study provided information on the age, sex, educational qualifications, income range, year of HIV diagnosis, and ART information (Online Appendix 1).

#### Health symptoms

The Medical Symptoms Questionnaire (MSQ) used in our study was modified from the MSQ compiled by the Institute for Functional Medicine (2020). It identifies symptoms that help diagnose underlying illness and tracks progress over time. Each symptom is based on the health profile of the patient over the previous 3 months. The scoring system is organised into 15 symptom-specific domains covering the head, eyes, ears, nose, mouth and throat, skin, heart, lungs, digestive tract, joints and muscles, weight, energy and activity, mind, emotions, and others. Respondents are required to assign a score ranging from 0 to 4 for 71 symptoms observed over the previous 14 days. A score of 0 means never having experienced such symptoms; 1–2 means occasional symptoms, not severe (‘1’) or severe (‘2’) effects; 3 and 4 mean frequent symptoms and not severe (‘3’) or severe (‘4’) effects. Therefore, a lower score means a lower symptom burden (Institute for Functional Medicine 2020). Although, the MSQ has yet to be validated and has inconsistent categorisation of items (Williams, Steinberg & Berzin 2021), a modified version, which is used in routine monitoring and evaluation at the study sites is adapted for the purpose of our study. This questionnaire asks participants to respond in ‘yes’ or ‘no’ to symptoms they have experienced over the last 14 days (Online Appendix 1).

#### Disability

The WHO Disability Assessment Schedule (WHODAS 2.0) tool was used to assess functioning and disability in various core activities essential in daily life (Üstün et al. 2010). The 12-item version is used for a brief assessment of overall functioning and asks questions about six domains: cognition (learning, concentrating), mobility (standing, walking), self-care (washing, dressing), getting along (keeping friendships, dealing with people), life activities (work and school), and participation (joining community activities, emotional effects). Individuals complete the WHODAS by answering each question on a 5-point Likert scale (range: 0–4), with higher scores indicating increasing difficulty in completing the task (Online Appendix 1). The WHODAS calculates ‘simple’ and ‘complex’ sum scores. The scores assigned to each item are summed (range: 0–48) in ‘simple’ scoring, with higher scores indicating greater disability (Paton & Lane 2020). Multiple levels of difficulty are factored for each item in ‘complex’ or item response theory-based scoring using a downloadable scoring sheet from the WHODAS website, providing a disability range of 0 (no disability) to 100 (total disability) (Paton & Lane 2020). The tool has been validated and demonstrated to be effective for screening functionality and disability among PLWH (Barbosa 2020). Furthermore, the internal reliability of the entire WHODAS 2.0 using Cronbach α is 0.89 (Holmberg et al. 2021).

The first author and two research assistants collected participant data according to the study protocol requirements. All questionnaires were in English language, and participants who required additional help were assisted in completing the questionnaire.

### Operational definitions

Human immunodeficiency virus-related disability was defined as any PLWH with a score of one or more on the WHODAS 2.0 (Kietrys et al. 2019). The term, ‘HIV-related disability’, used in our study refers to the physical, mental, social, and functional limitations experienced by PLWH (O’Brien et al. 2008). This term is commonly used in rehabilitation contexts to distinguish between individuals with milder challenges who can be treated simplistically and those with more severe conditions that require advanced rehabilitative therapy (Brown, 2022).

### Data analysis

The data from the completed questionnaires were captured in Microsoft Excel and imported into IBM^®^ Statistical Package for the Social Sciences (SPSS^®^) 25 (IBM Corp. 2017) for analysis. Descriptive statistics were applied to all the demographic variables. Quantitative data and the categorical variables were described in terms of mean ± standard deviation (*M* ± s.d.) and frequency or percentage, respectively. Odds ratio (OR) and confident interval (CI) were used to determine the strength of association between dependent and independent variables. Binary logistic regression analysis was performed to rank the relative importance of exposure variables with outcome variables. The level of significance was set at *p* < 0.05 for all the analyses. To determine which variables were independently associated with the main outcomes of WHODAS disability, all variables were entered into the univariate and multivariate regression models. Variables with *p* value less than 0.25 in the final model were considered as statistically significant.

### Ethical considerations

Approval was obtained from the Human Research Ethics Committee of the University of the Witwatersrand (M200906) and the Lagos State University Teaching Hospital Health Research Ethics Committee (LREC/06/10/1547). The clinical director of the participating testing and treatment centres granted permission. Written informed consent was obtained from the participants.

## Results

### Socio-demographic characteristics

The socio-demographic characteristics of the participants are presented in Table 1. The mean age of the participants was 42.2 ± 10.43 years, with a range of 22 to 70 years. Majority of the participants were females (*n* = 281; 73%), married (*n* = 219; 56.9%), had a secondary education (*n* = 194, 50.4%), and earned less than the national minimum wage of ₦30 000 (*n* = 221; 59.3%).

**TABLE 1:** Socio-demographic characteristics of the participants (*N* = 385).

Socio-demographic characteristics	*n*	%
**Age (years)**		
18–30	52	13.5
31–50	252	65.5
51 and above	81	21.0
**Gender**		
Men	104	27.0
Women	281	73.0
**Marital status**		
Married	219	56.9
Single	101	26.2
Widowed or Divorced or Separated	65	16.9
**Education**		
No formal education	24	6.3
Primary school	51	13.3
Secondary school	194	50.4
Post-secondary	115	29.9
**Income per month (₦)**		
Less than ₦18 000	113	29.4
₦18 001 – ₦30 000	115	29.9
₦30 001 – ₦70 000	108	28.1
Above ₦70 000	49	12.7

### Human immunodeficiency virus profile and medical symptoms

Table 2 outlines the years since diagnosis, and their current medical symptoms. All participants were on ART (100%). With regards to HIV diagnosis, the mean (s.d.) years since diagnosis was 7.52 ± 4.57 with 80% people being diagnosed in the second decade of the 2000s (2011–2020). All participants were on ART. Most respondents experienced headaches, weight loss, and muscular pain in the last 14 days (36.6%, 27.8% and 16.9% respectively).

**TABLE 2:** Human immunodeficiency virus information and medical symptoms (*N* = 385).

Variables	*n*	%	Mean	s.d.
**HIV information**				
Year since diagnosis	-	-	7.52	4.57
**Medical symptoms**				
Confusion	26	6.7	-	-
Breathlessness	17	4.4	-	-
Fatigue	61	15.8	-	-
Diarrhoea	26	6.8	-	-
Nausea and vomiting	16	4.2	-	-
Headache	141	36.6	-	-
Stomach pain	34	11.2	-	-
Change in taste, sore mouth	23	6.0	-	-
Skin itching or changes	65	16.9	-	-
Muscular pain	107	27.8	-	-
Fever	66	16.9	-	-
Weight loss	125	32.5	-	-

Note: Owing to a poor CD_4_ cell count and viral load monitoring, most participants did not know their actual CD_4_ cell count and viral load. Thus, on account of so much missing data, the CD_4_ cell count was not analysed.

HIV, human immunodeficiency virus; s.d., standard deviation.

### Human immunodeficiency virus-related disabilities of the participants

**FIGURE 1:**
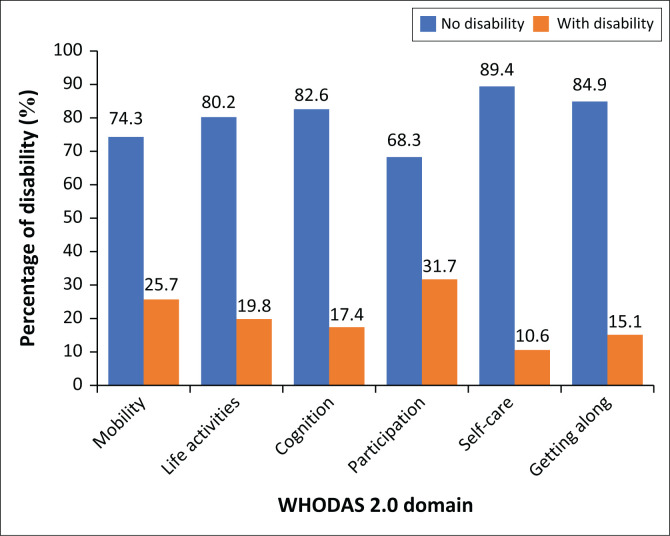
Human immunodeficiency virus-related disability of the participants by WHODAS 2.0 domain (*N* = 385).

Disability in this sample of PLWH stayed below what was expected based on previous literature. Figure 1 provides information on the pattern of disability grouped by domain and the frequency as measured by the WHODAS 2.0 questionnaire. Overall, 152 (39.5%) of the participants scored 1 or more on the WHODAS 2.0 questionnaire. Participants experienced the most disability in the participation domain (31.75) and had the least disability in self-care domain (10.6%) experiencing disability with functioning and participation in activities of daily living (see Online Appendix 2, Table 1-A2).

### Socio-demographic characteristics of participants with and without disability

Table 3 shows the association between the socio-demographic characteristics of PLWH and their disability status (i.e. with or without disability). The result shows that only marital status (χ^2^ = 9.467, *p* = 0.09) and education level (χ^2^ = 15.669, *p* = 0.01) had significant association with disability status.

**TABLE 3:** Association between socio-demographic profiles and disability level.

Socio-demographic characteristics	With disability (*N* = 152)		Without disability (*N* = 233)		χ^2^	*p*
	*n*	%	*n*	%		
**Age (years)**					6.104	0.191
18–30	27	17.8	25	10.7		
31–50	89	58.6	162	69.5		
51 and above	36	23.7	46	19.7		
**Gender**					0.234	0.629
Men	39	25.7	65	27.9		
Women	113	74.3	168	72.1		
**Marital status**					9.467	0.009*
Married	87	57.2	132	56.7		
Single	49	32.2	52	22.3		
Widowed or separated	16	10.5	49	21.0		
**Education**					15.669	0.001*
No formal education	8	5.3	16	6.9		
Primary school	20	13.2	31	13.3		
Secondary school	61	40.1	133	57.1		
Post-secondary	62	40.8	53	22.7		
**Income per month (₦)**					4.523	0.210
Less than ₦18 000	50	32.9	56	24.0		
₦18 000 – ₦30 000	41	27.0	74	31.8		
₦30 000 – ₦70 000	40	26.3	68	29.2		
Above ₦70 000	16	10.5	33	14.2		

* *p* < 0.05, statistically significant.

### Associated factors of HIV-related disability among people living with HIV

All socio-demographic variables in the univariable analysis whose chi-square significant level is ≤ 0.25 are included in the multivariable analysis (binary logistic) (Table 4). The results show that individuals aged 51 and above have statistically significantly higher odds of HIV-related disability compared to those aged 18–30 years (AOR: 1.6 [1.4, 3.5]). Single and widowed or separated individuals (AOR: 2.2 [1.1, 4.2]; AOR: 3.4 [1.5, 7.7]), and individuals with an income of ₦18 000 or less (AOR = 3.9, 95% CI [1.7, 8.9]) have statistically significantly higher odds of HIV-related disability compared to those with income above N70 000.

**TABLE 4:** Multilevel regression models of factors associated with HIV-related disability (*N* = 385).

Variable	COR	95% CI	AOR	95% CI
**Age (in years)**				
18–30	1.0	-	1.0	-
31–50	1.08	04, 3.2	0.5	0.7, 1.6
51 and above	0.8	0.3, 1.8	1.6	1.4, 3.5
**Marital status**				
Married	1.0		1.0	
Single	2.0	1.1, 3.8	2.2	1.1, 4.2
Widowed or separated	2.9	1.5, 5.7	3.4	1.5, 7.7
**Education**				
No formal education	1.0	-	1.0	-
Primary school	0.4	0.2, 1.08	0.2	0.1, 0.7
Secondary school	0.6	0.3, 1.1	0.5	0.2, 1.0
Post-secondary	0.4	0.2, 0.6	0.3	0.2, 0.6
**Income per month (₦)**				
Less than ₦18 000	1.8	0.9, 3.7	3.9	1.7, 8.9
₦18 000 – ₦30 000	1.1	0.6, 2.3	2.0	0.9, 4.4
₦30 000 – ₦70 000	1.2	0.6, 2.5	1.9	0.9, 4.0
Above ₦70 000	1.0	-	1.0	-

AOR, adjusted odds ratio; CI, confidence interval; COR, crude odds ratio.

## Discussion

Our study showed that the prevalence of disability among PLWH in Nigeria is 39.5%. This figure is notably lower than the 51.9% (South Africa) and 79.5% (United Kingdom) reported by Brown et al. (2022), utilising the same outcome measures. While the reason for this large disparity is unclear, one potential explanation, as discerned in studies employing self-reported measuring techniques, could be the inclination for overestimating one’s abilities (Akashi-Ronquest et al. 2011; Mactaggart et al. 2016). Additionally, an individual’s perception of disability is a vital construct that could influence the variation in reported disability levels (Babik & Gardner 2021). On a local scale, the identified prevalence in this study stands lower than that reported in Ibadan, Nigeria, where 71.1% of participants exhibited mild to extreme disabilities (Olaleye et al. 2017). Similar to the findings of Olaleye et al. (2017), the highest level of disability in our study was reported in the participation domain, possibly indicative of a shift in the disability experience of PLWH from concerns related to physical well-being to challenges in daily life activities. Consequently, the limitations confronted by PLWH in this study are predominantly associated with their interaction within their environment.

Participation restriction among PLWH may be influenced by several socio-demographic factors such as age, gender, education level, and income level. For example, the mean age of participants was 42.2 ± 10.43 years, with a range of 22 years to 70 years. These statistics are similar to those related to the mean age of participants in studies conducted in Nigeria, such as Olaleye et al. (2017) and Folasire, Irabor and Folasire (2012), who reported mean ages of 38.1 ± 9.0 years and 35.4 ± 7.0 years, respectively. Although there was no significant association between age and disability in our study, the educational level and marital status showed a significant association (*p* = 0.001 and *p* = 0.009, respectively). This could possibly be explained by female vulnerability to HIV in Nigeria which is further increased by the existence of a patriarchal society that promotes gender inequality and the disproportionate distribution of education, which limits female access to socio-economic benefits (Oyekale 2014). Kposowa (2013) suggested a relationship between HIV prevalence and marital status as divorced, separated, or widowed women had a higher risk of HIV infection in their study than single, married, or cohabiting women (Kposowa 2013).

Also, the feminisation of the HIV and AIDS epidemic remains a national and global problem, as evidenced by the gender gap distribution in this study and other similar studies conducted in Nigeria (Awofala & Ogundele 2018; Chiwuba 2022; Iyayi et al. 2011). In North Central Nigeria, 58% of the participants in the study by Ahmed et al. (2022) were females. Additionally, Ezechi et al. (2016) reported that the prevalence of women living with HIV in Southwest Nigeria increased from 60.2% in 2004 to 68.6% in 2015. Findings revealed that most participants were married (56.7%), 26.2% were single, and 19.6% were widowed, separated, or divorced. The female preponderance observed aligns with the sex distribution observed in other studies from sub-Saharan Africa (Karim & Baxter 2019) and globally, where over half (54%) of all individuals with HIV worldwide are females (UNAIDS 2023). A possible explanation could also be the observation that men are less inclined than women to participate in studies, thus skewing the results (Ryan et al. 2019). In regions where customary and religious laws heavily subjugate women’s rights, the preponderance of gender-based violence, particularly toward married women, contributes to the spread of HIV infection (Orisaremi 2017).

Regarding income level, although there was no significant association between disability and income level, considering that the estimated financial cost per individual living with HIV is ₦N36 065 in direct private healthcare expenses and indirect income loss (Ndukwe et al. 2018), the socio-economic impact of HIV cannot be overlooked. Despite the minimum wage in Nigeria being increased from ₦18 000 to ₦30 000 in April 2019 (Urama 2019), more than half (59.3%) of the sample population earned less than ₦30 000 ($65.07) monthly. On the contrary, the relationship between educational level and disability was shown to be statistically significant, demonstrating a greater prevalence of impairment among those with lower educational attainment compared to those with higher educational attainment. These findings are similar to prior literature (Olaleye et al. 2017) and support the hypothesis that people with greater levels of education may benefit from increased healthcare accessibility, which may be affected by higher employment and earning capability. Furthermore, it is possible that people with higher education levels are more likely to make health-conscious decisions, proactively treating symptoms that may develop to impairment.

More than 65% of the respondents had been diagnosed with HIV in the previous 7 years, and the results showed that all respondents were on ART. This indicates that Nigeria is on the path to achieving the Joint United Nations Program on HIV/AIDS 95-95-95 targets. Human immunodeficiency virus continues to affect the immune system and presents various symptoms at different stages of infection. People living with HIV who start ART early have an improved total life expectancy (Katz & Maughan-Brown 2017). Respondents from this study reported medical symptoms experienced in the previous 14 days such as headache, fever, fatigue, and muscular pain, all of which concur with the findings of other authors (Schreiner et al. 2020; Zhu et al. 2021). While most findings in literature (O’Brien et al. 2008; Olaleye et al. 2017) report association between CD4 count and disability, this study did not obtain information on the CD4 count and its relationship with disability level among PLWH as most participants did not have accurate records of these values, and thus was unable to be analysed.

### Strengths and limitations

The participants’ self-reported experiences of disability and symptoms over a period of 30 days were certain to include recall bias, which may not be accurate. Similarly, the cross-sectional design adopted in this study demands that causality between the explanatory variables and disability cannot be inferred. However, despite these limitations, the study’s strengths indicate that it has scientific and practical implications for policy and practice in HIV care. These findings could also have translational relevance in clinical practice and the management of disability in PLWH.

## Conclusion

The prevalence of disability among PLWH receiving ART was 39.5% in this study, with more than one-third of participants experiencing some form of disability. This highlights the significant prevalence of disability in this population, providing important insights into its prevalence and socio-demographic correlates in the Nigerian context. The findings shed light on the nuanced factors that shape disability experiences and highlight the need for tailored interventions that take into account the unique challenges that PLWH face. It is imperative to advocate for continuous disability screening, identification of potential risk factors, and development of effective management strategies. Recognising disability as a potential indicator of health status and quality of life, a longitudinal study to establish a cause-effect relationship between HIV-related disability and presumed risk factors is recommended. Furthermore, future research should consider the efficacy of various interventions aimed at improving the quality of life for PLWH, contributing to a better understanding of the disease and improving health outcomes in this population.
